# Jugular Venous Response for Risk Stratification in Heart Failure

**DOI:** 10.7759/cureus.58423

**Published:** 2024-04-16

**Authors:** Masaki Noguchi, Kenichi Kasai, Sakiko Honda, Chieko Sakai, Kuniyasu Harimoto, Tatsuya Kawasaki

**Affiliations:** 1 Department of Cardiology, Matsushita Memorial Hospital, Moriguchi, JPN; 2 Department of Rehabilitation, Matsushita Memorial Hospital, Moriguchi, JPN

**Keywords:** response, prognosis, physical examination, jugular vein, heart failure

## Abstract

Background: The response of jugular venous pressure (JVP) to increased preload with inspiration has been recognized as a method of stratifying risk in the management of heart failure (HF). Whether the JVP response to inspiration may be more effective than other simple approaches in this setting remains unclear.

Methods: This study enrolled 79 patients with stable HF. JVP was assessed from the right internal jugular vein in the sitting position and was considered high if visible above the right clavicle at rest. JVP responses to inspiration, the five-repetition sit-to-stand test (5-STS), and squatting were also evaluated. The primary outcome was a composite of all-cause death and hospitalization for worsening HF.

Results: JVP assessment after 5-STS and during squatting was not conducted in two and 14 HF patients, respectively, due to physical limitations. During a mean follow-up of 837 days, the primary outcome was associated with a high JVP at rest (hazard ratio, 2.47; 95% confidence interval [CI], 1.09 to 5.60; P <0.05), with inspiration (hazard ratio, 2.53; 95% CI, 1.17 to 5.46; P <0.05), after 5-STS (hazard ratio, 2.61; 95% CI, 1.23 to 5.97; P <0.05), and during squatting (hazard ratio, 2.40; 95% CI, 1.03 to 6.06; P <0.05). Among patients without a high JVP at rest, the specificity of the primary outcome at one year was greater for the JVP response to inspiration (89%) and squatting (92%) than for the response to 5-STS (80%).

Conclusions: JVP response to increased preload with inspiration may be a simple and practical method for risk assessment in patients with stable HF.

## Introduction

Assessment of jugular venous pressure (JVP) is essential in the management of heart failure (HF) [1,2]. Unfortunately, this physical examination has often been neglected in clinical practice because of its complicated methodology, e.g., having to assess the vertical distance measurement between the sternal angle and the top of the jugular vein at the 45˚ position [3,4]. This traditional method may not apply to patients with acute decompensated HF in the emergency setting due to their symptoms, such as orthopnea. In addition, it is difficult to assess JVP response with this traditional method, although not only the rest of the findings but also its responses are imperative in patients with chronic heart disease, as shown by exercise electrocardiography and dobutamine echocardiography.

More recently, the simple assessment of JVP, in which the right internal jugular vein above the right clavicle is visible or not in the sitting position [5], has gained attention. We have reported that the response of JVP to inspiration or Kussmaul's sign can detect even HF patients with a slightly high JVP who might be missed by the original simple method [6]. Also, we have shown that risk stratification of HF patients using JVP response to inspiration is independent of patient characteristics, such as left ventricular ejection fraction or the presence or absence of atrial fibrillation [7]. The underlying mechanism can be explained by increased preload with inspiration, but whether other approaches, including increased afterload and cardiac workload, may be more effective in this setting remains unclear. Therefore, the objective of the present study was to compare JVP response to inspiration with other simple approaches for risk stratification in patients with HF.

## Materials and methods

This study included 79 patients with HF who were treated at Matsushita Memorial Hospital between January 2020 and July 2020. Eligible patients were 18 years of age or older and had been stable in the outpatient clinic for more than six months. The diagnosis of HF was made according to the guidelines for the diagnosis and treatment of acute and chronic HF [8]. Ethical approval was obtained from Matsushita Memorial Hospital, and informed consent was obtained from all patients.

Patient characteristics, including age, sex, body mass index, presence of comorbidities (hypertension, diabetes, dyslipidemia, ischemic heart disease, atrial fibrillation, and implantable cardiac devices), and medications (beta-blockers, angiotensin-converting enzyme inhibitors/angiotensin receptor blockers, mineralocorticoid receptor antagonists, sodium-glucose cotransporter-2 inhibitors, angiotensin receptor neprilysin inhibitors, and diuretics) were investigated. Plasma brain natriuretic peptide (BNP), hemoglobin, albumin levels, and estimated glomerular filtration rate were also examined.

JVP assessment

**Figure 1:**
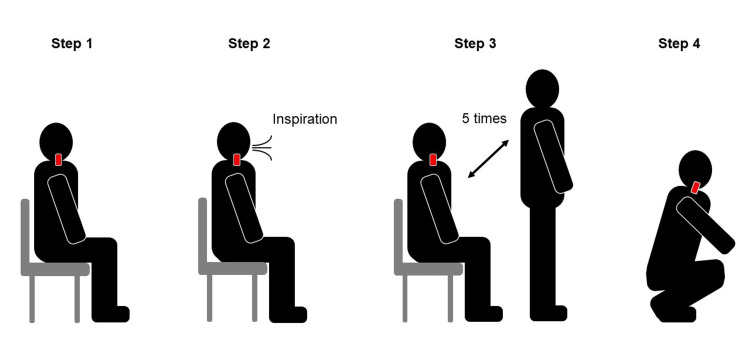
JVP assessment Step 1. JVP is assessed at rest in the sitting position and was considered high when the right internal jugular venous pulsation was visually identified above the right clavicle (Red bar). Step 2. The response of JVP to inspiration is assessed. If the response of JVP to inspiration remained equivocal, breath-holding for a few seconds was added. Step 3. The JVP response to standing up and sitting down quickly five times is evaluated. Step 4. The JVP response to squatting is finally evaluated.

JVP was assessed at rest in the sitting position, as previously described [6,7]. Briefly, JVP was considered high when the right internal jugular venous pulsation was visually identified above the right clavicle (Video 1). The response of JVP to inspiration, which was elicited by repeated full inspiratory effort at a normal respiratory rate without breath-holding, forced respiratory effort, or Valsalva, was also assessed (Figure 1). If the response of JVP to inspiration remained equivocal, breath-holding for a few seconds was added. Although the external jugular vein is easier to visualize than the internal jugular vein, the internal jugular vein on the right side was assessed because of its features, including its larger size and lack of competent valves with a straight line to the superior vena cava and right atrium. In addition, the JVP response to the five-repetition sit-to-stand test (5-STS), i.e., standing up and sitting down quickly five times without using the hands to push up from the chair [9], and the JVP response to squatting while allowing the use of the hands, such as holding onto a table or chair, were evaluated.

**Video 1:** A representative case of high JVP Note that the right internal jugular venous pulsation is visually identified above the right clavicle at rest in the sitting position.

Echocardiography

Echocardiography was performed using the Vivid E9 (GE Healthcare, Milwaukee, WI). Measurements included left ventricular ejection fraction, left ventricular end-diastolic diameter, left ventricular mass index, and left atrial volume index. The maximum and minimum diameters of the inferior vena cava at rest and during inspiration were also measured.

Follow-up and endpoint

All patients were followed up after the assessment of JVP responses. Patient information was obtained from available medical records and interviews with the patients and/or their treating physicians. The primary outcome was a composite of all-cause death and hospitalization for worsening HF, requiring the initiation of intravenous treatment with inotropic, vasodilator, and/or diuretic treatment and mechanical ventilation or circulatory support [6,7].

Statistical analysis

Categorical variables were compared using the chi-square test. Continuous variables were expressed as the mean ± standard deviation and were compared using the Student’s t-test. Time-to-event data were evaluated with the use of Kaplan-Meier estimates and Cox proportional hazards models, stratified by JVP response to each approach. Cox models were used to calculate hazard ratios, 95% confidence intervals (CI), and p-values. A two-sided p <0.05 was considered statistically significant.

## Results

This study included 79 patients with HF (mean age, 72 years; 52 men). The main causes of HF were ischemic heart disease in 19 patients (24%), valvular heart disease in 17 (22%), dilated cardiomyopathy in 15 (19%), and hypertrophic cardiomyopathy in 12 (15%).

Of all patients, 12 (15%) had a high JVP at rest, and the remaining 67 (85%) had no high JVP at rest. Assessment of JVP response to 5-STS and squatting was not conducted in two patients (3%) and 14 patients (18%), respectively, due to physical limitations such as knee or back pain. Of all the patients studied, a high JVP was observed in 33 patients (42%) with inspiration, 26 (33%) after 5-STS, and 40 (50%) during squatting. Among 12 patients with a high JVP at rest, all patients had a high JVP with inspiration, after 5-STS, and during squatting, except one with no high JVP after 5-STS. Of 67 patients without a high JVP at rest, 21 showed a high JVP with inspiration, 14 showed a high JVP after 5-STS, and 31 showed a high JVP during squatting.

There were no significant differences in baseline characteristics between patients with a high JVP and patients without a high JVP as assessed by each method except for the incidence of atrial fibrillation (Table 1). On echocardiographic findings, the left atrial volume index and the diameters of the inferior vena cava were significantly higher in patients with a high JVP than those in patients without a high JVP, as assessed by most methods.

**Table 1:** Characteristics of the patients. Data are expressed as mean ± standard deviation or number (%). ACE: angiotensin-converting enzyme; ARB: angiotensin receptor blocker; ARNI: angiotensin receptor neprilysin inhibitor; BNP: brain natriuretic peptide; eGFR: estimated glomerular filtration rate; JVP: jugular venous pressure; MRA: mineralocorticoid receptor antagonist; SGLT2: sodium-glucose cotransporter 2; 5-STS: five-repetition sit-to-stand test. *P <0.05, **p <0.01 versus patients without a high JVP at rest; #p <0.05, ##p <0.01 versus patients without a high JVP with inspiration; †p <0.01 versus patients without a high JVP after 5-STS; ‡p <0.05 versus patients without a high JVP during squatting.

	All patients (n=79)	High JVP			
		Rest (n=12)	Inspiration (n=33)	5-STS (n=26)	Squatting (n=40)
Age, years	71.7±11	74.2±9.1	75.4±9.2	75.6±8.1	73.7±9.0
Male	53 (67%)	7 (58%)	21 (64%)	16 (62%)	27 (68%)
Body mass index, kg/m^2^	22.4±3.7	20.4±3.8	21.5±3.5	21.4±3.5	22.0±3.9
Comorbidities					
Hypertension	41 (52%)	8 (67%)	22 (67%)	14 (54%)	22 (55%)
Diabetes	28 (35%)	3 (25%)	15 (45%)	12 (46%)	14 (35%)
Dyslipidemia	33 (42%)	6 (50%)	16 (48%)	12 (46%)	16 (40%)
Ischemic heart diseases	24 (30%)	3 (25%)	9 (27%)	8 (31%)	11 (28%)
Atrial fibrillation	34 (43%)	9 (75%)*	22 (67%)^##^	15 (58%)	23 (58%)^‡^
Pacing device implantation	10 (13%)	1 (8%)	3 (9%)	4 (15%)	6 (15%)
Medications					
Beta-blockers	44 (56%)	8 (67%)	18 (55%)	19 (73%)	23 (58%)
ACE inhibitors/ARBs	52 (66%)	7 (58%)	19 (58%)	17 (65%)	27 (68%)
MRAs	21 (27%)	2 (17%)	8 (24%)	8 (31%)	13 (33%)
SGLT2 inhibitors	26 (33%)	3 (25%)	12 (36%)	9 (35%)	16 (40%)
ARNI	11 (14%)	2 (17%)	4 (12%)	5 (19%)	8 (20%)
Diuretics	50 (63%)	6 (50%)	21 (64%)	12 (46%)	23 (58%)
Laboratory data					
BNP, pg/mL	189±236	271±211	187±157	221±180	203±195
Hemoglobin, g/dL	13.5±2.0	13.2±2.2	13.2±2.5	13.3±2.2	13.4±2.2
Albumin, g/dL	4.0±0.5	3.8±0.4	3.9±0.4	3.9±0.5	3.9±0.5
eGFR, mL/min/1.73m^2^	53.3±16.9	49.2±22.4	51.5±19.9	49.3±19.4	53.0±18.5
Echocardiographic findings					
LV ejection fraction, %	51.7±15.7	54.8±15.7	54.7±15.0	49.5±16.2	51.2±17.5
LV end-diastolic diameter, mm	49.7±9.5	46.8±11.1	48.2±9.7	49.5±10.7	50.0±10.1
LV mass index, g/m^2^	109±27	119±29	111±32	111±27	111±28
Left atrial volume index, ml/m^2^	60.8±39.7	93.9±77.9	75.8±53.4^#^	75.4±58.3	71.5±50.7^‡^
Inferior vena cava					
Maximum diameter, mm	15.1±4.9	19.5±5.4**	17.4±4.5^#^	17.2±4.9^†^	16.6±4.4^‡^
Minimum diameter, mm	8.3±4.1	11.2±5.9	10.1±4.9^#^	9.9±5.3^†^	9.6±4.1^‡^

**Figure 2:**
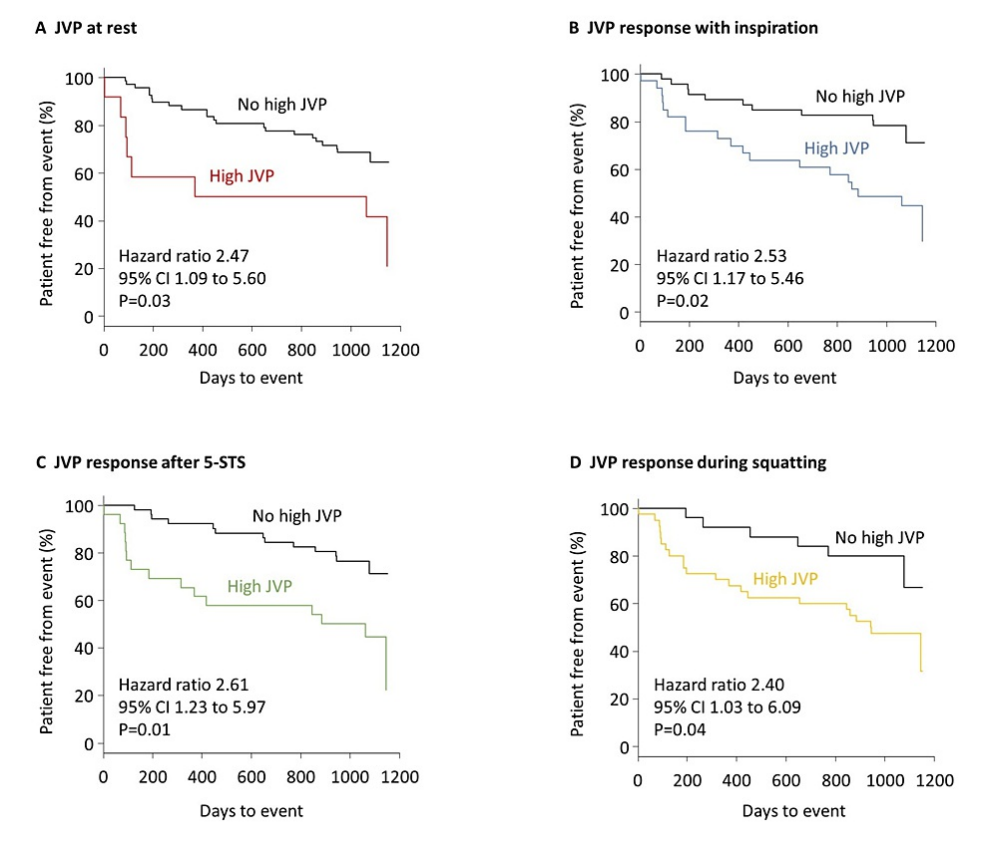
Time-to-event analysis The primary outcome was a composite of all-cause death and hospitalization for worsening heart failure. Kaplan-Meier curves for the primary outcome are shown in all patients according to jugular venous pressure (JVP) findings at rest (A), with inspiration (B), after the five-repetition sit-to-stand test (5-STS) (C), and squatting (D).

**Figure 3:**
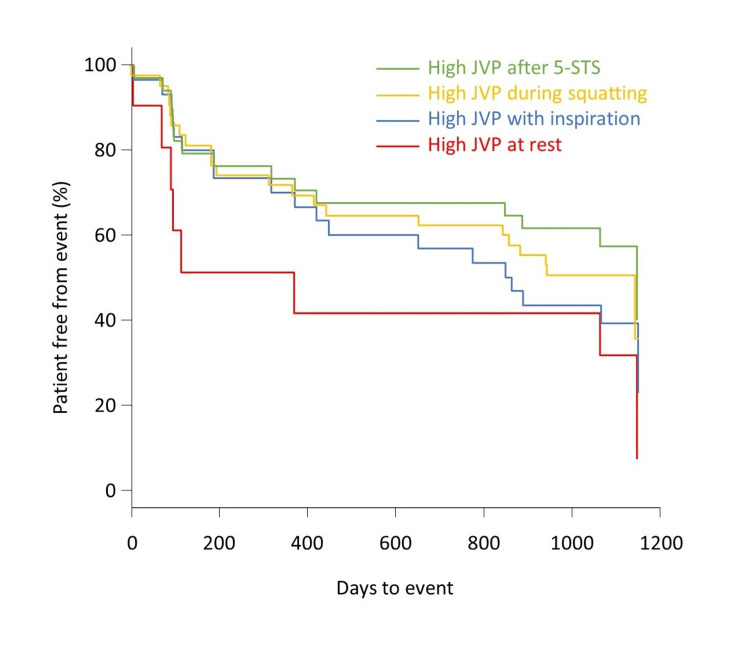
Methodological comparison Kaplan-Meier curves for the primary outcome are shown for patients with a high JVP as assessed by each approach. Note that these groups partially overlap because some patients had positive results on two or more approaches for the high JVP assessment.

During a mean follow-up of 837 days, 30 patients developed a primary outcome event: six died and 24 were hospitalized for worsening HF. The primary outcome was associated with a high JVP at rest (hazard ratio, 2.47; 95% CI, 1.09 to 5.60; p <0.05), with inspiration (hazard ratio, 2.53; 95% CI, 1.17 to 5.46; p <0.05), after 5-STS (hazard ratio, 2.61; 95% CI, 1.23 to 5.97; p <0.05), and during squatting (hazard ratio, 2.40; 95% CI, 1.03 to 6.06; p <0.05) (Figure 2). The event-free curve for the primary outcome was lowest in patients with a high JVP at rest, followed by patients with a high JVP with inspiration and during squatting, and highest in patients with a high JVP after 5-STS (Figure 3), although statistical analyses were not performed because of the partial overlap of patients in each group. Among all 67 patients without a high JVP at rest, the specificity of the primary outcome at one year was greater for the JVP response to inspiration (89%) and squatting (92%) than for the response to 5-STS (80%), although the sensitivities were similarly low (26%, 23%, and 28%, respectively).

## Discussion

We assessed JVP at rest and in response to three approaches (i.e., with inspiration, after 5-STS, and during squatting) in 79 patients with stable HF. A high JVP was observed in 12 patients at rest, 33 with inspiration, 26 after 5-STS, and 40 during squatting. The primary outcome defined as a composite of all-cause death and hospitalization for worsening HF was significantly associated not only with a high JVP at rest but also with inspiration, after 5-STS, and during squatting. The hazard ratios for the JVP responses to the three approaches were almost the same, around 2.5. Still, the specificity of the primary outcome at one year was greater for the JVP response to inspiration and squatting than for 5-STS in patients without a high JVP at rest.

In this study, three simple approaches were used to examine the predictive values of risk stratification in patients with stable HF. It is worth noting that each approach differs significantly in how the heart is loaded. For example, increased preload plays a major role in the effect of inspiration on the heart, as negative pleural pressure with inspiration causes increased right ventricular volume due to increased venous return or preload [10]. Given that squatting from a standing position increases not only preload but also arterial blood pressure or afterload [11], this positional change would be more useful to avoid missing a slightly elevated JVP in patients with HF. Indeed, in the current study, more patients showed a high JVP during squatting than with inspiration, but the hazard ratios and specificities of the primary outcome at one year were almost the same between the JVP responses to inspiration and squatting. Furthermore, the fact that squatting could not be performed in all patients suggests that a JVP response to inspiration would be more practical for risk stratification of HF.

The effect of 5-STS on the heart needs to be elucidated in terms of preload and afterload, but it is intuitive to assume that 5-STS leads to increased cardiac workload. In a study of elderly patients hospitalized in an intensive care unit [12], 5-STS led to increases in heart rate (i.e., from 79.7 ± 10.2 bpm to 86.6 ± 9.7 bpm, p = 0.001) and systolic blood pressure (i.e., from 118 ± 21.4 mmHg to 129 ± 21.5 mmHg, p = 0.031). However, in the current study, the specificity of the primary outcome was lower for the JVP response to 5-STS than for the JVP response to inspiration. The exact underlying mechanism remains unclear, but an insufficient increase in cardiac workload or double product, an index of myocardial oxygen consumption [13], after 5-STS may explain our findings. We can safely assume that the response of JVP to inspiration for HF risk stratification would be preferred for HF risk stratification. This speculation may be supported by the fact that all patients underwent JVP response to inspiration, whereas 5-STS could not be performed in some patients, albeit a small number, due to physical limitations.

The mechanism by which the presence of a high JVP was significantly associated with cardiac events in patients with HF, regardless of the approaches used in our study, remains uncertain. However, our results are intuitive because a high JVP is one of the most useful physical findings in the assessment of ventricular filling pressures [14]. Again, it is worth noting that JVP reflects not only right-sided pressures but also left-sided filling pressures in patients with HF [1]. Drazner et al. examined the relationship between right atrial pressure and pulmonary capillary wedge pressure in 4,079 patients before heart transplantation and found concordant hemodynamics of the two indexes (i.e., both being elevated or not, defined as right atrial pressure ≥10 mmHg and pulmonary capillary wedge pressure ≥22 mmHg, respectively) in around three-quarters of the patients [15].

The current study has several limitations. It was conducted at a single center with a small number of selected patients, which may not be generalizable to the global HF population. The predictive values of a high JVP were mainly driven by rehospitalization for worsening HF. Our study may be underpowered to detect the predictive power of survival; larger studies with a longer follow-up period are warranted. Patients with a slightly elevated JVP may be missed even with the addition of inspiration, but these patients are unlikely to be at high risk for cardiac events. Other assessments may be more useful, such as hepatojugular reflux, which was not performed in this study.

## Conclusions

JVP responses to inspiration, 5-STS, and squatting assessed in the sitting position were useful approaches as a risk stratification in patients with stable HF. Given the facts that JVP assessment after 5-STS and during squatting was not conducted in some patients due to physical limitations and that the specificity of the primary outcome at one year was greater for the JVP response to inspiration and squatting than for the response to 5-STS, the JVP response to increased preload with inspiration may be the most practical approach in this setting.
